# Habitat and Vegetation Variables Are Not Enough When Predicting Tick Populations in the Southeastern United States

**DOI:** 10.1371/journal.pone.0144092

**Published:** 2015-12-11

**Authors:** R. T. Trout Fryxell, J. E. Moore, M. D. Collins, Y. Kwon, S. R. Jean-Philippe, S. M. Schaeffer, A. Odoi, M. Kennedy, A. E. Houston

**Affiliations:** 1 Department of Entomology and Plant Pathology, University of Tennessee, Knoxville, Tennessee, United States of America; 2 Department of Biology, Christian Brothers University, 650 East Parkway South, Memphis, Tennessee, United States of America; 3 Department of Biology, Rhodes College, 2000 North Parkway, Memphis, Tennessee, United States of America; 4 Department of Earth Sciences, University of Memphis, Memphis, Tennessee, United States of America; 5 Department of Forestry, Wildlife and Fisheries, University of Tennessee, Knoxville, Tennessee, United States of America; 6 Department of Biosystems Engineering and Soil Science, University of Tennessee, Knoxville, Tennessee, United States of America; 7 Department of Biomedical and Diagnostic Sciences, University of Tennessee, Knoxville, Tennessee, United States of America; 8 Department of Biology, University of Memphis, Memphis, Tennessee, United States of America; University of North Dakota School of Medicine and Health Sciences, UNITED STATES

## Abstract

Two tick-borne diseases with expanding case and vector distributions are ehrlichiosis (transmitted by *Amblyomma americanum*) and rickettiosis (transmitted by *A*. *maculatum* and *Dermacentor variabilis*). There is a critical need to identify the specific habitats where each of these species is likely to be encountered to classify and pinpoint risk areas. Consequently, an in-depth tick prevalence study was conducted on the dominant ticks in the southeast. Vegetation, soil, and remote sensing data were used to test the hypothesis that habitat and vegetation variables can predict tick abundances. No variables were significant predictors of *A*. *americanum* adult and nymph tick abundance, and no clustering was evident because this species was found throughout the study area. For *A*. *maculatum* adult tick abundance was predicted by NDVI and by the interaction between habitat type and plant diversity; two significant population clusters were identified in a heterogeneous area suitable for quail habitat. For *D*. *variabilis* no environmental variables were significant predictors of adult abundance; however, *D*. *variabilis* collections clustered in three significant areas best described as agriculture areas with defined edges. This study identified few landscape and vegetation variables associated with tick presence. While some variables were significantly associated with tick populations, the amount of explained variation was not useful for predicting reliably where ticks occur; consequently, additional research that includes multiple sampling seasons and locations throughout the southeast are warranted. This low amount of explained variation may also be due to the use of hosts for dispersal, and potentially to other abiotic and biotic variables. Host species play a large role in the establishment, maintenance, and dispersal of a tick species, as well as the maintenance of disease cycles, dispersal to new areas, and identification of risk areas.

## Introduction

The roles of *Amblyomma americanum* (lone star tick), *Amblyomma maculatum* (Gulf coast tick), and *Dermacentor variabilis* (American dog tick) in tick-borne disease (TBD) transmission has been directed at host association studies [1–6], but field studies investigating habitat use and niche partitioning where these species co-exist are severely lacking. Currently, these tick species’ distributions overlap with one another [5–8]. What makes these species of particular interest is the fact that they transmit a number of pathogens that affect humans and animals. *Amblyomma americanum* is a competent vector of *Ehrlichia ewingii* and *E*. *chaffeensis* causing ehrlichiosis [9–11], of *Francisella tularensis* causing tularemia [12, 13], and of the newly identified Heartland virus [14]. *Amblyomma maculatum* is the vector of *R*. *parkeri* causing American Boutonouse fever [11, 15] and *E*. *ruminantium* causing heartwater in ruminants [16]. *Dermacentor variabilis* is the vector of agents causing spotted fever group Rickettsiae and is a known vector of *R*. *rickettsii* [17, 18]. Within the southeastern United States both *Ehrlichia* and *Rickettsia* diagnoses are increasing, likely due, in part, to increasing tick numbers and expanding ranges [19].

Integrated tick management (ITM) programs include the application of acaricides to animals and vegetation, and various methods of habitat disturbance [20]. Acaricides alone are often not effective long-term when applied to existing vegetation because tick populations re-establish quickly as hosts move through the habitat; however, vegetation removal along with acaricides can produce conditions not suitable for tick survival and establishment [21]. Mechanical clearing of vegetation has been shown to result in an immediate reduction of local tick populations, though long-term reduction was not demonstrated [22]. Reduced *A*. *americanum* populations have been associated with landscape alterations including mechanical removal of vegetation such as clearing undergrowth and overstory, which reduce relative humidity and soil moisture [20]. Various results have been obtained in experiments that examined controlled burning as a means of tick control. Hoch et al. [23] found that controlled burning of woodlots was not effective in long-term control of *A*. *americanum* ticks, while other studies observed short-term reductions in the populations of some species [22, 24, 25]. Successful habitat treatments and a sustained lowering of tick populations are not single application procedures; as these sites become neglected, natural plant succession occurs and the site generally reverts to previous conditions.

Both abiotic and biotic components of the environment influence tick populations [26–28]. Because vegetation type influences the presence and movement of host species, it is likely that vegetative composition and structure are related to the presence, density, or persistence of tick species and to the probability of successful host acquisition. Microclimates and associated environmental habitats have been reported for a number of ticks; and these variables include humidity, temperature, and day light hours [26, 27]. For example, ticks will desiccate if isolated from microclimates with high temperatures [21]. The larval stage of ticks is the stage most susceptible to desiccation, and questing activity is “self”-restricted to periods when potential for desiccation is reduced [29]. After brief periods of questing on vegetation for passing hosts, the potential for desiccation forces the tick to travel back into the leaf litter [30]. Some ticks have adapted to longer questing periods and hotter temperatures. *Dermacentor variabilis* is considered desiccation-tolerant, *I*. *scapularis* can directly absorb fluids from saturated and mildly sub-saturated atmospheres, and *Amblyomma* ticks have a thickened waxy coating on their cuticles to prevent water loss [30]. It is often assumed that most ticks prefer secondary growth woodland habitats with a dense understory [26, 27], but these studies did not directly compare adjacent habitats with equal host opportunities. *Ixodes scapularis* populations have been associated with deciduous, dry to mesic forests and alfisol-type soils of sandy or loam-sand textures overlying sedimentary rock [28], and these data have been used to identify potential locations with Lyme disease (aka risk areas). Thus, the habitat’s microclimate, vegetation, and soil type likely have significant effects on tick abundance and questing activity and on the dynamics of TBDs. Habitat suitability also includes other stages in a tick’s life, including overwintering, molting, and oviposition sites.

Previous work at Ames Plantation in southwestern Tennessee and within the previously identified Rocky Mountain Spotted fever hot spot [31] during the summer of 2012 indicated that *A*. *americanum* abundance was positively associated with increasing basal area and ground cover [7]. Additionally, *A*. *americanum* specimens were identified with three *Ehrlichia* species (*E*. *ewingii*, Panola Mountain *Ehrlichia*, *E*. *chaffeensis*) and *Anaplasma odocoilei*. While these infected ticks were collected from a variety of habitats, positive collections were primarily in summer months (June), and no spatial clustering of positive ticks was evident [7]. Interestingly, *Ehrlichia* and *Anaplasma* were detected only in sites where both *A*. *americanum* and *D*. *variabilis* were present [7]. Additionally at that site, *R*. *parkeri* was identified in questing and host-collected *A*. *maculatum* (*unpublished*). Since the questing site is the location where pathogen transmission begins [32], it is essential to identify questing locations to minimize tick encounters and subsequent tick bites. There is a critical need to identify the landscape and vegetation features where each tick species is likely encountered to classify and pinpoint risk areas. Consequently, an in-depth tick prevalence study focused on the ticks of the southeastern United States was conducted during June 2014 when TBD cases peak in Tennessee [33, 34].

The objectives of this study were to specify the summer questing habitats of ticks commonly encountered in the southeastern United States through classification of vegetative and landscape characteristics. Additionally, we attempted to identify indicator plant species that inferred the presence of one or more tick species. The overarching hypothesis was that habitat and vegetation variables that influence questing behavior and host availability predict tick abundances. Identifying preferred habitats for questing ticks is the first step for an overall ITM program, which then includes identification of infected-questing habitats to study disease transmission, identify management options, and help with disease diagnosis.

## Materials and Methods

### Site Selection

**Fig 1:**
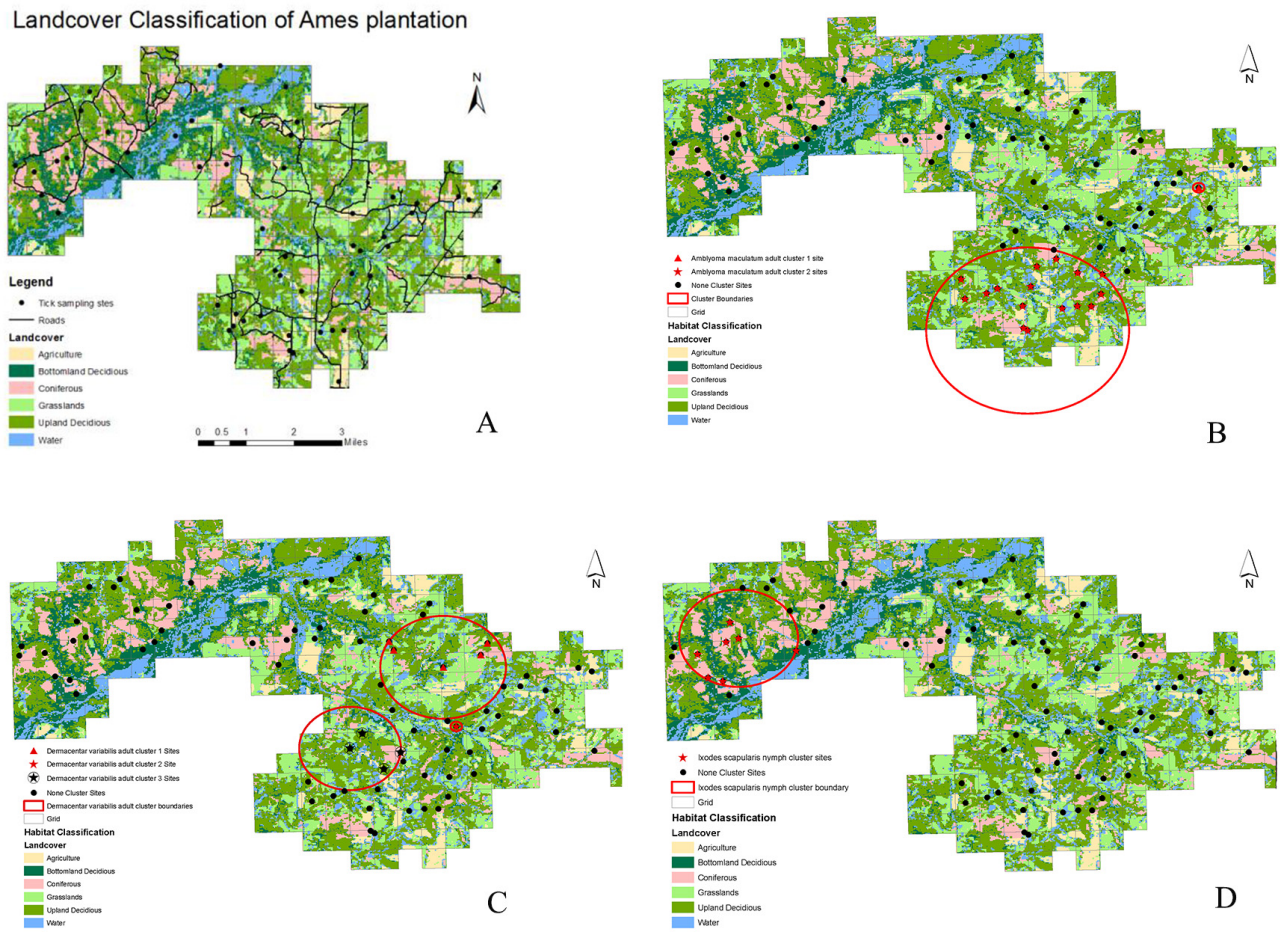
Ticks were collected from a variety of land cover classifications (A), and spatial clustering analysis indicated *Amblyomma maculatum* adults had two clusters (B), *Dermacentor variabilis* adults had three clusters (C), and *Ixodes scapularis* nymphs had one cluster (D). Land cover classification is generated from the Landsat 8 OLI image downloaded at http://earthobservatory.nasa.gov

Ames Plantation (35.115366 N, -89.216735 W) is a University of Tennessee-managed research center in western Tennessee. Permission was obtained from the director, R. Carlisle, and coauthor A. Houston to use the site. It is a 7,446 hectare contiguous tract (74.5 km^2^) containing an array of land-use types, including commodity row crops (e.g., cotton, soybean, corn, grain, wheat), pastures for horses and beef cattle, native warm season grasslands, and forests, including loblolly pine plantations, bottomland hardwoods, and upland hardwoods. These land uses are underlain with a broad spectrum of physiography, ranging from mesic bottomlands associated with the North Fork of the Wolf River to xeric upland sites, which provides significantly different ecological systems and suitable habitats for a number of different animals, including potential tick hosts. From the Ames’ Quality Deer Management Program, which includes an observation and harvest grid system with each grid ~40.5 ha, 76 tick-sampling sites were randomly stratified. Sites included 15 bottomland deciduous sites, 28 upland deciduous sites, 15 coniferous sites, and 18 open grasslands (Fig 1). Each of the 76 sites contained one plot, 100m long by 20m wide. Three 100m transects were placed side-by-side in these plots, 10m apart. Sampling from multiple sites provided an unbiased and representative proportion of the different environmental variables.

### Tick Collection

Based upon a trapping methods comparison (*unpublished*), CO_2_-dry ice traps, conventional dragging, CO_2_-dragging, and CO_2_-flagging were used for tick sampling during June of 2014 when tick-borne diseases peak in Tennessee [7, 33, 34]. One dry ice trap was set at the middle of each center 100m transect and left overnight. The remaining three collection methods were randomly assigned to each 100m transect and checked for ticks every 20m along each transect. All encountered ticks were removed, counted, and placed in vials of 80% ethanol. Ticks were identified in the laboratory to life-stage, species, and sex [35–37].

### Vegetation Characterization

Vegetation was sampled in a 1m^2^ quadrat at the center point of each tick drag along the center transect at each site. Therefore, plant community composition and structure were sampled at 10, 30, 50, 70, and 90m along each centerline. Within each sampling area, each plant species was identified and percent cover of each species was visually estimated with an adapted Daubenmire cover scale (<1%, 1–5%, 5–10%, 10–25%, 25–50%, 50–75%, 75–95%, > 95%) and transformed to median values for community similarity analyses. Transect data for each site were combined for both diversity and composition pattern analyses. To determine percentage canopy openness and leaf area index (LAI), hemispherical photographs taken in the transect center (50m) with a fisheye lens on a 1m tripod were analyzed with Gap Light Analyzer software [38]. All photographs were taken on cloudless days in late May between 0830 and 1330 h. To determine vertical structure, basal area was estimated using a handheld prism, identifying and including tree species with a diameter at breast height (dbh) > 5cm at each sampling point along the transect. All basal area data were combined for each site for analysis.

### Landscape Characterization

At each site, three-soil core samples were taken every 5m along the middle transect. Soil samples were combined together to produce one composite sample for each 20m segment of the transect, and placed in a single labeled bag. Samples were stored on ice, brought back to the laboratory, and stored in a freezer at -20°C until analyzed. In the laboratory, soil pH was determined by mixing soil and deionized water in a 1:1 volume by weight ratio, shaking samples, and allowing them to settle for an hour before reading pH using a pH meter [39].

Remote Sensing and GIS methods were used to characterize four general site properties: 1) Normalized Difference Vegetation Index (NDVI as proxy for photosynthetic activity), 2) proximity to roads, 3) proximity to water body and 4) patchiness of land cover. Landsat 8 OLI scene (WRS-2 Path 22 Row 36) taken on April 9, 2014 was used for land cover classification and calculation of NDVI. The vegetation survey for 76 tick-sampling sites and high-resolution aerial photograph from ESRI World Imagery were used to create training sites for the six land cover types of water/marsh, agriculture, grass/pasture, bottomland hardwoods, upland hardwoods, and coniferous plantation. In addition, a field survey was conducted during mid-July 2014 across Ames Plantation and a total of 84 GPS validation points were identified on the ground when tick-borne diseases peak in Tennessee [7, 33, 34]. Land cover classification was then carried out using per-pixel classification methods and an error matrix was also created. NDVI was calculated using Landsat red and near-infrared regions of spectral reflectance as NIR-Red/NIR+Red to indicate the overall photosynthetic activity level of the surface [40]. Because proximity of study sites to roads may influence the presence of ticks as a major CO_2_ source (as an attractant) or as a site for road kill (where ticks would detach) [41], roads found within the boundary of Ames Plantation were identified as secondary roads, local roads, four-wheel-drive roads, service roads, and private roads. Many tick-sampling sites were located close to several roads, so we applied a weighted overlay method in grid analysis to give larger and more frequently used roads a larger weight when calculating the proximity value. The weight values are from AEH’s knowledge at Ames Plantation as secondary roads were weighted at 50%, local roads weighted at 20%, and the remaining three types were weighted at 10% each. The Ames Plantation landscape has several kinds of water features, such as creeks, lakes, and marshes. Because proximity of study sites to different kinds of water bodies may relate to tick abundance [42] the Euclidian distance was calculated from each study site to the closest water body as a proximity to water. Patchiness of land cover (mixed type of land covers vs. single types), or the spatial complexity of the environment, was calculated by summing the edges of the land cover classification polygons within a 300m buffer around each study site.

### Statistical Analysis

Habitat types and the presence of each tick species were compared with contingency tests (*X* ^*2*^ tests), and habitat preference was determined with an ANOVA on log (x+1) transformed tick counts from each site. To determine the relationship between soil pH, vegetation characteristics, landscape measures, and each tick’s presence, a multivariate analyses with a significance level of α = 0.05 was used. A PCA was generated to visualize how sites and habitat groups differed with respect to the different predictor variables, but not to examine their relationship (S1 Fig). ANOVA models consisted of continuous (e.g., pH, vegetation height) and habitat type as a categorical variable. For tick species that had multiple life stages represented in five or more sites (*A*. *americanum* nymphs and adults), MANOVA was used to examine habitat use. For MANOVAs, the dependent variable was the number of individuals in each stage class (adults or nymphs) at a site. For tick species in which only adults were well represented (*A*. *maculatum* and *D*. *variabilis*), an ANOVA was used with number of adult ticks at a site as the dependent variable. Dependent variables were log-transformed to meet model assumptions. In both MANOVA and ANOVA, our predictor variables were habitat type, plant diversity (Shannon Index), plant evenness (E_h_), basal area, canopy openness, soil pH, NDVI, distance to water, distance to roads, and patchiness. We tested all continuous predictor variables for an interaction with habitat type. While *I*. *scapuarlis* was collected, a model was not built for *I*. *scapularis* because it occurred in only four sites and did not meet the five or more site minimum. Datasets are provided in S1 Table.

### Cluster Detection and Identification

Spatial scan statistics, implemented in SaTScan [43] were used to detect locations of statistically significant spatial clusters of collected tick species and life stages. The spatial scan statistic uses a circular window of variable radius that moves across the study area. The radius of the window was set to vary from 0 up to a maximum value that included 50% of the population under investigation [44]. As the window of the statistic moves across the study area (Ames Plantation), it defines a set of different neighboring sampling sites each of which is a candidate for a potential cluster [45]. Clusters are assessed by comparing the number of specimens (adults, nymphs, or total) within the window with the number that would be expected if the specimens were randomly distributed in the study area. The test of significance of identified clusters is based on a likelihood ratio test whose *P*-value is obtained through Monte Carlo testing. Clusters were assessed under the discrete Poisson model assumption and 999 Monte Carlo replications were used for significance testing. The null hypothesis of no clusters was rejected when the simulation *P* ≤ 0.05. Results of cluster detection were then imported into ArcGIS 10.1 [46], and the spatial distribution of identified clusters and sites was displayed in maps.

## Results

### Tick Collection

A total of 5050 ticks were collected in June 2014 from the 76 sites consisting of 4904 (97.11%) *A*. *americanum*, 128 (2.53%) *D*. *variabilis*, 11 (0.22%) *A*. *maculatum*, and 7 (0.14%) *I*. *scapularis* (Table 1). Of the *A*. *americanum*, 4166 (84.95%) were nymphs, 387 (7.89%) were adult females, 340 (6.93%) were adult males, and 11 (0.22%) were larvae. The remaining ticks were 71 *D*. *variabilis* females, 57 *D*. *variabilis* males, 9 *A*. *maculatum* males, and 2 *A*. *maculatum* females. Additional *A*. *americanum* larvae were encountered throughout the study by the investigators, but were not included in the analysis because the investigators could not guarantee where each was acquired due to size for detection and walking into sites.

**Table 1:** A total of 5050 ticks were collected from 76 sites consisting of bottomland deciduous, upland deciduous, coniferous, and grassland habitats. A majority of the specimens were *Amblyomma americanum*, *A*. *maculatum* nymphs, *D*. *variabilis* nymphs, and *I*. *scapularis* adults were not collected.

Habitat (no. of sites)	No. ticks collected (mean ± SEM)					
	*Amblyomma americanum*		*Amblyomma maculatum*	*Dermacentor variabilis*	*Ixodes scapularis*	Total
	Nymph	Adult	Adult	Adult	Nymph	All life stages
Bottomland Deciduous (n = 15)	233 (15.53 ± 5.005)	77 (2.57 ± 0.717)	0 (NA)	29 (0.97 ± 0.398)	1 (0.07 ± 0.067)	340 (22.67 ± 6.035)
Upland Deciduous (n = 28)	2837 (101.32 ± 48.122)	358 (6.39 ± 1.406)	0 (NA)	52 (0.93 ± 0.264)	5 (0.18 ± 0.146)	3262 (116.5 ± 49.940)
Coniferous (n = 15)	913 (60.87 ± 20.186)	238 (7.93 ± 1.188)	0 (NA)	32 (1.07 ± 0.339)	1 (0.07 ± 0.067)	1185 (79.0 ± 21.400)
Grasslands (n = 18)	183 (10.17 ± 2.795)	54 (1.50 ± 0.384)	11 (0.31 ± 0.158)	15 (0.42 ± 0.163)	0 (NA)	263 (14.61 ± 3.343)
Total (n = 76)	4166 (54.82 ± 18.563)	727 (4.78 ± 0.657)	11 (0.07 ± 0.040)	128 (0.84 ± 0.148)	7 (0.09 ± 0.057)	5050 (66.45 ± 19.390)

*An additional 11 larvae were recorded from upland deciduous (10 specimens) and a coniferous site (1 specimen).

From the 76 sites sampled, a mean of 66.5 (± 19.39) specimens were collected (range: 1 to 1216 specimens). Twenty-four (31.6%) sites had only 1 species (23 sites had only *A*. *americanum* and 1 site had only *D*. *variabilis*), 46 (60.5%) sites had 2 species (at all of these sites *A*. *americanum* was the dominant species), and 6 (7.9%) sites had 3 species. Four of the sites with three tick species were grassland sites with *A*. *americanum*, *A*. *maculatum*, and *D*. *variabilis*. The remaining two sites were upland deciduous and harbored *A*. *americanum*, *D*. *variabilis*, and *I*. *scapularis*. Collections from forested sites (bottomland deciduous, upland deciduous, and coniferous) were similar to one another and were primarily composed of *A*. *americanum*, *D*. *variabilis*, and *I*. *scapularis*. In contrast, grassland sites did not have any *I*. *scapularis* and were the only sites where *A*. *maculatum* were collected. *Amblyomma americanum* and *D*. *variabilis* were also collected at grassland sites.

**Fig 2:**
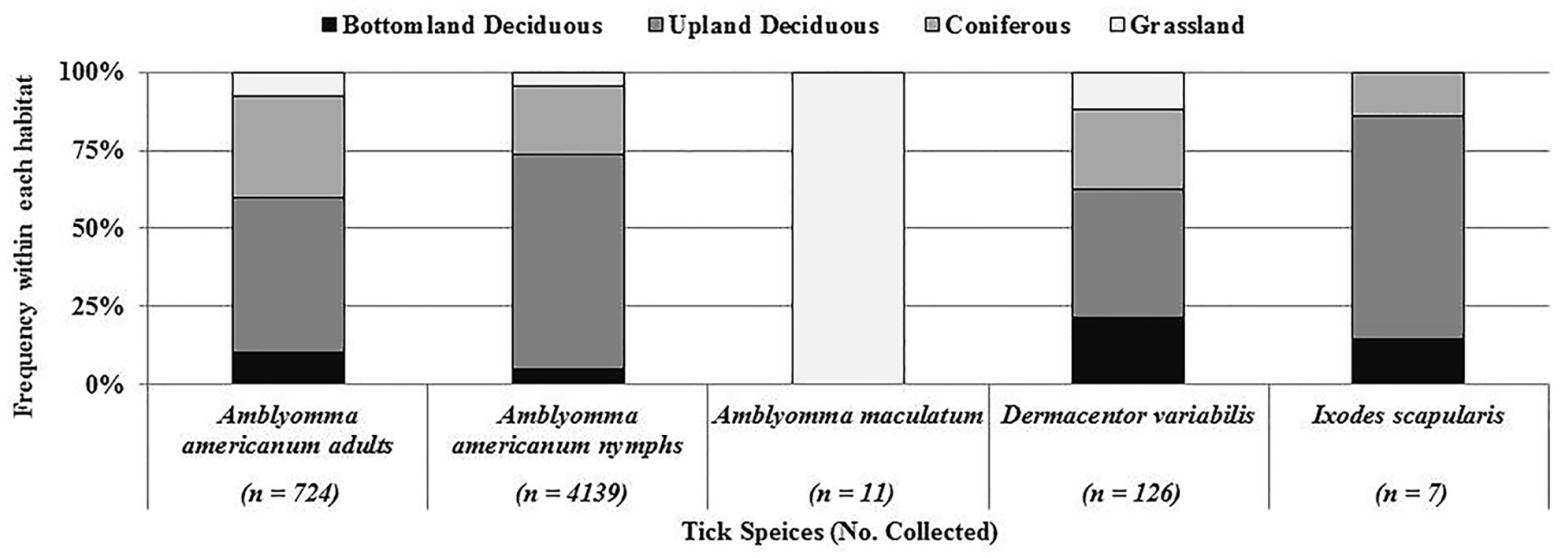
Frequency of each tick species and life stage (number collected) among the different habitat types.

Different habitats were significantly associated with the presence of each tick species (represented by means) (Table 1, Fig 2). At one upland deciduous site, 1186 *A*. *americanum* nymphs were collected, which was 518 more specimens than the next population dense site. Population size indicated by mean abundance suggested significantly more ticks were collected from coniferous sites (79.0 ± 82.90) than grassland sites (14.6 ± 3.34), but not upland deciduous sites (116.5 ± 49.94). Bottomland deciduous sites (22.7 ± 6.04) were not significantly different from either upland deciduous or grassland sites (*F* = 6.71; df = 3, 72; *P* = 0.0005). The presence of *A*. *americanum* did not differ among habitats (*X* ^*2*^ = 4.12; df = 3; *P* = 0.249), but population size indicated by abundance did suggest significantly more *A*. *americanum* were collected from upland deciduous (114.5 ± 49.94) and coniferous (76.8 ± 21.06) sites, than from bottomland deciduous (20.7 ± 5.95), and those collections were significantly greater than grassland sites (13.2 ± 3.38) (*F* = 7.26; df = 3, 72; *P* = 0.0003). The presence of *A*. *maculatum* was significantly different across habitats (*X* ^*2*^ = 24.8; df = 3, 72; *P* < 0.001), as all 11 specimens were collected from grassland sites (0.6 ± 0.22). The presence of *D*. *variabilis* did not differ between habitats (*X* ^*2*^ = 4.54; df = 3; *P* = 0.209), with 1.9 ± 0.73 ticks in bottomland deciduous, 1.9 ± 0.47 in upland deciduous, 2.1 ± 0.58 in coniferous, and 0.8 ± 0.28 in grassland sites. The presence of *I*. *scapularis* did not differ across habitats (*X* ^*2*^ = 1.32; df = 3; *P* = 0.725), and neither did population sizes (*F* = 0.4724; df = 3, 72; *P* = 0.7025).

Comparisons of *A*. *americanum* life stages mirrored total *A*. *americanum* collections. A total of 727 adults were collected of which significantly more were collected at coniferous sites (15.9 ± 2.12) and upland deciduous sites (12.8 ± 2.79) than bottomland deciduous sites (5.1 ± 1.30) and grassland sites (3.0 ± 0.72) (*F* = 12.34; df = 3, 72; *P* < 0.0001). This pattern was also true for the 4166 nymphs collected from the 76 sites as coniferous sites (60.9 ± 20.19) had significantly more ticks than grassland sites (10.2 ± 2.80), but not more than upland deciduous sites (101.3 ± 48.12) or bottomland deciduous sites (15.5 ± 5.01) (*F* = 3.2112; df = 3, 72; *P* = 0.028).

### Vegetation Characterization

There were no differences among site types for any predictor variables including Shannon-Weiner Diversity index (*F* = 1.146; df = 3, 72; *P* = 0.366), evenness (*F* = 0.928; df = 3, 72; *P* = 0.432), leaf area index (*F* = 1.595; df = 3, 72; *P* = 0.198), openness (*F* = 2.092; df = 3, 72; *P* = 0.109), and basal area (*F* = 1.427; df = 3, 72; *P* = 0.242). PCA also showed that these predictor variables do not group based on habitat type (S1 Fig); this is likely an artifact of habitat site designations. A Detrended Correspondence Analysis (DCA) was conducted to determine whether habitat types were distinguishable based on vegetation measures (S2 Fig). DCA indicated two vegetation habitat types (grassland and forest) instead of four (grassland, bottomland deciduous, upland deciduous, and coniferous) (S2 Fig). To keep consistent with tick sampling protocol and other predictor variables, we conducted our analyses using four habitat types (upland deciduous, bottomland deciduous, coniferous, and grassland). For all predictor variables there were no significant pairwise Tukey comparisons.

### Landscape Characterization

Landcover classification (Fig 1) conducted with maximum-likelihood methods indicated that Ames Plantation consists of agriculture (7%), bottomland deciduous (14%), grasslands/pastures (19%), coniferous (12%), upland deciduous (32%), and water/marsh (12%). The overall classification accuracy against 160 ground truth data (76 tick-sampling sites and 84 ground-truth points) was 70% and Kappa value—accuracy that taking a random chance into account—was 62% (Table 2). The largest classification confusion occurred between bottomland deciduous and upland deciduous cover type as these two have similar canopy reflectance values from satellite imagery (error of commission or percentage of misclassified pixel against ground truth data, was 0.48 for bottom land deciduous and 0.31 for upland deciduous). Mean NDVI values for all 76 tick-sampling sites was 0.26, while overall NDVI for Ames plantation was 0.18 which reflected negative NDVI values for water bodies. The closest site to water was within 10m while the furthest site from a water body was located 1.3 km away. Mean distance to water was 313m (SD = 247m). Distance to roads calculated by weighted average by different road types is a unit less measure and produced a mean of 2608 (SD = 1604). Mean patchiness was 9173m (SD = 2129m).

**Table 2:** Error matrix for landcover classification in AMES plantation showed overall accuracy of 0.7 (Kappa value of 0.62) and the largest confusion found between bottomland deciduous and upland deciduous.

	Ground-truth land covers							
	Agriculture	Bottomland Deciduous	Grass/ Pasture	Coniferous	Upland Deciduous	Water/ marsh	Total	*Error of Commission
Agriculture	21	3	4	0	1	1	30	0.30
Bottomland Deciduous	3	17	2	4	7	0	33	0.48
Grass/pasture	1	1	14	0	0	1	17	0.17
Coniferous	1	2	0	20	4	1	28	0.28
Upland Deciduous	1	5	1	3	22	0	32	0.31
Water/marsh	0	0	1	0	0	19	20	0.05
Total	27	28	22	27	34	22	160	
**Error of Omission	0.22	0.39	0.36	0.25	0.35	0.13		0.70

*Error of commission: percentage of misclassified pixel against ground truth data

**Error of omission: percentage of omitted pixel against ground truth data

### Models of Habitat Use

MANOVA for *A*. *americanum* indicates that no variables were significant predictors of adult and nymph tick abundance (Table 3). Adult *A*. *maculatum* tick abundance was predicted by NDVI (*F* = 5.07; df = 1, 35; *P* = 0.03) and by the interaction between habitat type and plant diversity (*F* = 3.75; df = 3, 35; *P* = 0.02); combined, these significant predictors account for 22% of the total Sum of Squares (Table 4). For *D*. *variabilis*, no variables were significant predictors of adult tick abundance (Table 5).

**Table 3:** MANOVA table for abundances of *Amblyomma americanum* adults and nymphs indicates that no variables were significant predictors of adult and nymph tick abundance.

Potential Predictor	Df	Pillai	Approximate *F* value	Num. Df	Den. Df	*P* value
habitat type	3	0.133553	0.8348	6	70	0.54717
plant diversity	1	0.122842	2.38078	2	34	0.10773
plant evenness	1	0.044821	0.79772	2	34	0.4586
basal area	1	0.074734	1.37309	2	34	0.26701
distance to roads	1	0.040149	0.71108	2	34	0.49827
Patchiness	1	0.031333	0.54989	2	34	0.58206
Openness	1	0.010384	0.17838	2	34	0.8374
NDVI	1	0.005285	0.09032	2	34	0.91386
soil pH	1	0.130813	2.5585	2	34	0.09224
Water	1	0.065209	1.18588	2	34	0.3178
habitat type: plant diversity	3	0.234116	1.54673	6	70	0.17591
habitat type: plant evenness	3	0.05912	0.35537	6	70	0.90444
habitat type: basal area	3	0.157268	0.99569	6	70	0.43517
habitat type: distance to roads	3	0.109011	0.67255	6	70	0.6721
habitat type: patchiness	3	0.025544	0.15094	6	70	0.98829
habitat type: openness	3	0.214825	1.40395	6	70	0.22541
habitat type: NDVI	3	0.123006	0.76456	6	70	0.60023
habitat type: soil pH	3	0.088152	0.53793	6	70	0.77761
habitat type: water	3	0.118362	0.73388	6	70	0.62398
Residuals	35					

**Table 4:** ANOVA table for abundances of *Amblyomma maculatum* adults indicate *A*. *maculatum* presence can be predicated by NDVI and by interaction between habitat type and plant diversity.

Potential Predictor	Df	Sum Sq	Mean Sq	*F* value	*P* value
habitat type	3	0.164	0.0547	0.721	0.5463
plant diversity	1	0.0263	0.0263	0.346	0.5601
plant evenness	1	0.1009	0.1009	1.331	0.2565
basal area	1	0.1694	0.1694	2.234	0.1439
distance to roads	1	0.0101	0.0101	0.133	0.718
patchiness	1	0.006	0.006	0.079	0.7804
openness	1	0.0005	0.0005	0.007	0.9336
**NDVI**	1	0.3844	0.3844	5.068	0.0308*
soil pH	1	0.0041	0.0041	0.054	0.8172
water	1	0.0056	0.0056	0.074	0.7868
**habitat type: plant diversity**	3	0.853	0.2843	3.749	0.0195*
habitat type: plant evenness	3	0.1389	0.0463	0.611	0.6126
habitat type: basal area	3	0.0265	0.0088	0.117	0.9498
habitat type: distance to roads	3	0.3715	0.1238	1.633	0.1994
habitat type: patchiness	3	0.0427	0.0142	0.188	0.9041
habitat type: openness	3	0.4738	0.1579	2.082	0.1203
habitat type: NDVI	3	0.0119	0.004	0.053	0.9839
habitat type: soil pH	3	0.1233	0.0411	0.542	0.6568
habitat type: water	3	0.1516	0.0505	0.666	0.5784
Residuals	35	2.6543	0.0758		

Bolded values are significant (* *P* < 0.05).

**Table 5:** ANOVA table for abundances of *Dermacentor variabilis* adults indicate *D*. *variabilis* populations can be predicted by the interaction of habitat type and basal area.

Potential Predictor	Df	Sum Sq	Mean Sq	*F* value	*P* value
habitat type	3	0.577	0.1922	0.356	0.785
plant diversity	1	0.001	0.0008	0.001	0.97
plant evenness	1	0.021	0.0211	0.039	0.844
basal area	1	0.147	0.1471	0.273	0.605
distance to roads	1	0.325	0.325	0.602	0.443
patchiness	1	0.126	0.1256	0.233	0.632
openness	1	1.385	1.3848	2.567	0.118
NDVI	1	0.163	0.1632	0.303	0.586
soil pH	1	0.248	0.2478	0.459	0.502
water	1	0.012	0.0125	0.023	0.88
habitat type: plant diversity	3	0.103	0.0343	0.064	0.979
habitat type: plant evenness	3	0.113	0.0376	0.07	0.976
habitat type: basal area	3	2.877	0.959	1.778	0.169
habitat type: distance to roads	3	2.313	0.7711	1.429	0.251
habitat type: patchiness	3	1.643	0.5478	1.015	0.398
habitat type: openness	3	1.817	0.6057	1.123	0.353
habitat type: NDVI	3	2.913	0.9709	1.8	0.165
habitat type: soil pH	3	0.785	0.2618	0.485	0.695
habitat type: water	3	0.577	0.1924	0.357	0.785
Residuals	35	18.882	0.5395		

### Cluster Detection and Identification

Spatial clusters were not identified for *A*. *americanum* total counts, adult or nymphal counts indicating the species and life stages were found throughout Ames Plantation (*P* > 0.05). Analysis of *A*. *maculatum* adults identified two statistically significant clusters, one cluster with 1 site (*RR* = 64.6, *P* = 0.05) and a second cluster with sixteen sites (*RR* = 7.3; *P* = 0.028) (Fig 1b). Analysis of *D*. *variabilis* adults identified three statistically significant clusters, one cluster with five sites (*RR* = 3.1, *P* = 0.0005), a second cluster with one site (*RR* = 6.8, *P* = 0.0016), and a third cluster with four sites (*RR* = 3.1; *P* = 0.029) (Fig 1). While only seven *I*. *scapularis* were collected from four sites, these seven were found in a single statistically significant cluster (*RR* = 80.0, *P* = 0.001) (Fig 1d).

## Discussion

A thorough understanding of tick populations and their pathogens is essential to the accurate and timely diagnosis of TBDs, the development of risk assessments, and advancement of management plans to control ticks and reduce TBDs. This study identified few vegetation and landscape variables associated with tick presence or density, and this is likely due to ticks using hosts for dispersal and limiting our environmental variables to vegetation and landscape features to one tick season and study site. This study focused on vegetative and landscape features because these data are easier to obtain over broad geographic regions and because these traits likely influence ticks directly through abiotic factors and indirectly through their effects on host species. While some variables, such as NDVI and the interaction between habitat type and plant diversity, were significantly associated with one tick species, the amount of variation explained was low and not useful for predicting presence or density reliably. The NDVI coefficient was extremely small (9x10^-14^) and likely an artifact of the large number of 0s (tick absence); while statistically significant, the association is weak and not biologically useful for prediction. This low amount of variation explained by our models is likely due to the use of hosts for dispersal [47, 48] and potentially other environmental variables such relative humidity [49, 50] and soil conditions [28]. Host species play a large role in the establishment, maintenance, and dispersal of a tick species, as well as the maintenance of disease cycles and associated pathogens [29, 32, 51]. Identification of preferred host species for each tick species in western Tennessee will help determine the factors that aide in the establishment and maintenance of tick populations and identify potential mechanisms (i.e., host agents) of tick (and pathogen) dispersal.

Although we were unable to identify specific environmental variables associated with each tick species and/or life stage, we found significant spatial clusters for *A*. *maculatum*, *D*. *variabilis*, and *I*. *scapularis*. Again, we speculate that this clustering might be due to 1) uncharacterized environmental variables, 2) the need for additional seasonal sampling and replication, and 3) the use of hosts for dispersal. Other constraints that might favor or limit tick populations include the assemblage of host species and habitat parameters. This might include not just using hosts for dispersal and a food source, but also for “directed dispersal” to a suitable habitat that provides protection from the elements and permits successful molting to the next instar. The significant *I*. *scapularis* cluster was found in the western region of Ames, an area with established coniferous and deciduous stands, and habitat to a plentiful turkey and white-tailed deer population, both common *I*. *scapularis* hosts [6, 52, 53]. *Ixodes scapularis*, here found only in wooded areas of the plantation, likely spend a majority of its life in the shade of trees (both on the host and off the host in the environment). This tick is notably susceptible to desiccation and must use humidity to reabsorb fluids from the atmosphere [30]. The three *D*. *variabilis* clusters were located in the middle of the plantation, and this species is commonly collected from raccoons [2, 6, 54]. The location of the *D*. *variabilis* clusters is largely conventional agriculture with crops, field, and woods, but all with generally hard, clearly defined edges. For a cosmopolitan tick such as *D*. *variabilis*, which is considered desiccation-tolerant [30], conventional agricultural land represents an island habitat with habitat patches (heterogeneous in nature), but well defined with definite vegetative typing possible. For *Amblyomma* ticks with thickened waxy coatings on their epicuticle, host species were likely more responsible for distributions and clustering as *A*. *americanum* was found throughout the plantation (and use turkeys and white-tailed deer), and *A*. *maculatum* was collected only in small clusters that were defined as a heterogeneous blend of habitats. *Amblyomma maculatum* prefer to feed on a number of hosts including cattle and quail [29, 55], and the identified clusters in the southern region of Ames is home to the National Bird dog trials, where quail are released and habitat is managed for those quail. This area is a highly heterogeneous, patchy mix of woods, fields, and native grasslands. Together, these data and findings lead to additional questions about macro- and micro-scale parameters associated with tick presence and abundance. Ultimately, these spatial clusters provide useful information to guide future studies investigating factors responsible for the identified higher densities and could likely serve as sites for host trapping studies. Such studies could also include reasons for pathogen presence/absence at different sites, similar to work conducted by Ostfeld et al. [56] which identified small hosts with large populations and r-life histories as excellent reservoirs for *Borrelia burgdorferi*, *Babesia microti*, and *A*. *phagocytophilum*.

Due to the lack of significant environmental relationships, we have begun to examine host populations directly. Preliminary data on birds and small mammals were collected to identify tick-host-habitat relationships at the study site. Investigator Kennedy (unpublished) collected primarily the white-footed mouse (*Peromyscus leucopus*) from a variety of habitats with *I*. *scapularis*, *D*. *variabilis*, and *A*. *maculatum* as ectoparasites, and the hispid cotton rat (*Sigmodon hispidus*) only from grassland sites with *D*. *variabilis* and *A*. *maculatum* as ectoparasites. Investigator Collins (unpublished) mist-netted birds, and ticks were found on two individuals (0.65%). An adult female Indigo Bunting (*Passerina cyanea*) caught in a coniferous site had a rabbit tick (*Haemaphysalis leporispalustris*), and an adult female Carolina Wren (*Thryothorus ludovicianus*) netted in a bottomland hardwood site had two *Ixodes brunneus*. Both bird species nest near the ground (Indigo Buntings <1m and Carolina Wrens <2m) and both specimens were adult females with brood patches. Bird-habitat and small mammal-habitat associations were also identified; some host species were collected in all site types (white-footed mouse), while others were specific to open/grassland sites (hispid cotton rat, Indigo bunting).

It can be assumed from the literature [4–6, 35, 53–65] and from our findings that tick hosts have different tick communities, hosts have preferred habitats, and hosts often have a different ectoparasite community from questing collections (as *A*. *americanum* dominated questing collections and were absent from the host collections). Here, our observations might suggest that potential hosts nesting on or near the ground (e.g., mice, wrens) are more likely to harbor and transport ticks and TBDs than animals nesting or living in canopy settings (e.g., squirrels, nuthatches). Additionally, one could speculate that those animals (specifically birds) that migrate and nest near the ground could transport ticks further and introduce them to new areas. Nevertheless, future research into the use of niche-defined hosts and as indicators should be further investigated. Moreover, the patterns predicted by tick susceptibility to desiccation are likely correlated with host-habitat associations as well.

It is plausible that habitat use by potential hosts also varies across our field site. Generalist hosts, such as deer and turkeys, will use all four habitats, have a defined home range, and can be found throughout the plantation. It will be difficult to develop habitat models for ticks (such as *A*. *americanum*) that use these generalist hosts as primary hosts, so these tick species are either strongly influenced by a specific abiotic/biotic variable, or have the ability to use multiple habitats. Mesomammals such as raccoons, skunks, and opossums can also be found throughout the plantation, and these species have a generalized affinity for habitats, and there are likely some occasional seasonal tendencies, but few will be predictable. Ticks using these animals as primary hosts will probably be found in mixed environments, and true predictability with repeatability will be rare. Hosts with small home ranges, such as birds and small mammals, will likely have the greatest impact on tick populations. These animals commonly prefer specific vegetation types, and other abiotic/biotic parameters. As a part of the preliminary host studies, white-footed deer mice were collected in field, hardwood, and pine habitats; however, hispid cotton rats were only collected in field grass habitats. Hispid cotton rats are noted as primary hosts for immature *A*. *maculatum* [29, 55]. This leads us to hypothesize that host-habitat specificity will also likely influence tick presence and absence.

Although large numbers of ticks were collected, a majority of the questing ticks were *A*. *americanum*. The critical next step is to determine 1) how the environment influences a tick’s ability to locate a host, 2) how the environment influences the presence and abundance of potential tick hosts, and 3) how environmental variables and host community jointly influence population sizes and dispersal of each tick species (and subsequent pathogens). As with *I*. *scapularis* and Lyme disease, it is likely that the environment provides shelter and food sources for southeastern ticks with the ability to transmit *Ehrlichia* and/or *Rickettsia* pathogens. Moreover, the environment provides questing sites for tick attachment, and questing sites are essentially where pathogen transmission begins. Additional drivers into tick range expansion likely include climate warming and/or habitat change as both will affect the plant composition and subsequent host composition for ticks and their pathogens. Further studies into this system that include hosts, vectors, and pathogens [66] that describe the nidus of pathogen transmission [67] such as those presented by Simon et al. [68] are necessary for these southern TBDs.

These data serve as groundwork for commonly encountered ticks and for tick-habitat associations in the southeastern United States and demonstrate a need for 1) continued work on tick-habitat associations that include multiple seasons and sampling efforts, 2) inclusion of hosts in future studies, and 3) concurrent pathogen detection studies to identify areas with pathogen-infected ticks. These findings will assist future endeavors at field sites and serve as foundational data for tick distribution models for the region. Consequently, these findings serve as the basis for determining species distribution, identifying local tick habitats, and analyzing tick biological patterns. With additional tick and pathogen surveillance, these and additional data could contribute to preparing relative risk maps for both *Ehrlichia* and *Rickettsia* within the southeast. Identification of these sites assists administrators in developing practical and cost-effective strategies for tick control, so managers can monitor and treat areas with tick and infected-tick populations. The next step is to sample host communities (when ticks are actively questing and feeding), additional seasons (when other species and life stages are questing), and replicate the project over multiple years and sites to validate and expand on the model.

## Supporting Information

S1 Fig PCA results. PCA results depict how sites and habitat groups differed with respect to the different predictor variables, but not to examine their relationship.
(TIF)

S2 Fig DCA results. DCA results indicated habitat types were distinguishable based on vegetation measures; two vegetation habitat types (grassland and forest) instead of four (grassland, bottomland deciduous, upland deciduous, and coniferous).
(TIF)

S1 Table Datasets. Data files for MANOVA and ANOVA analyses.
(ZIP)
