# Supplementary figures and images for: Habitat and Vegetation Variables Are Not Enough When Predicting Tick Populations in the Southeastern United States
Source: PLoS One. 2015 Dec 11;10(12):e0144092. doi: 10.1371/journal.pone.0144092 (PMC4676690; doi:10.1371/journal.pone.0144092)

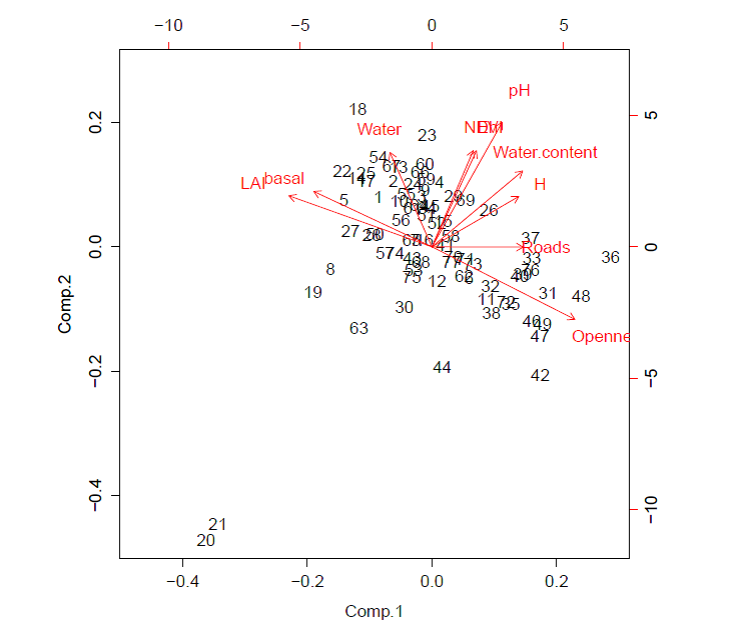

Supplement: S1 Fig — PCA results depict how sites and habitat groups differed with respect to the different predictor variables, but not to examine their relationship. (TIF) [file pone.0144092.s001.tif]

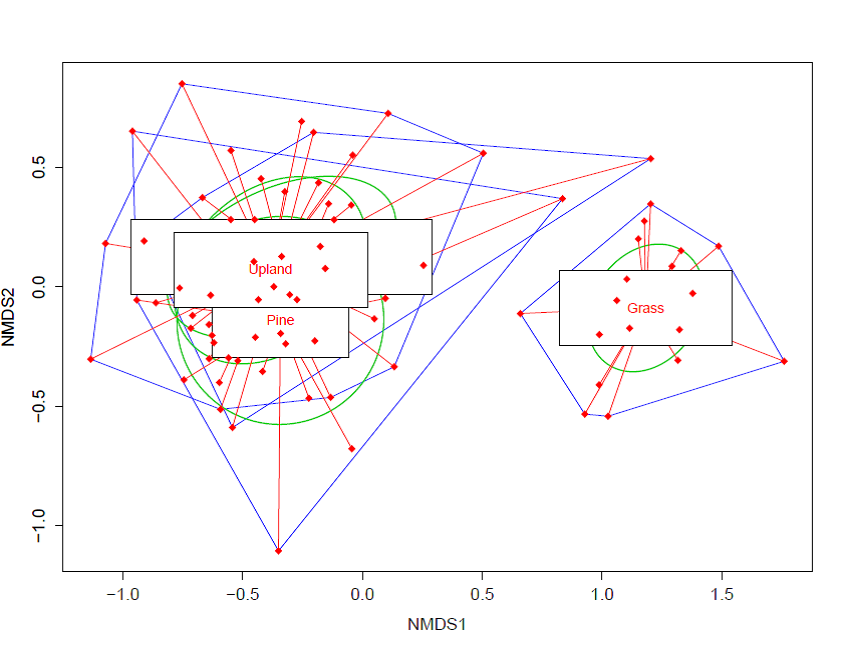

Supplement: S2 Fig — DCA results indicated habitat types were distinguishable based on vegetation measures; two vegetation habitat types (grassland and forest) instead of four (grassland, bottomland deciduous, upland deciduous, and coniferous). (TIF) [file pone.0144092.s002.tif]
